# Validation of the Collett-Lester fear of death scale in occupational therapy students: psychometric testing and implications for palliative care education

**DOI:** 10.1186/s12904-024-01386-7

**Published:** 2024-02-21

**Authors:** Daniel Gutiérrez-Sánchez, Inmaculada López-Leiva, Stella Martín-de-las-Heras, Leticia Rubio, Jaime Martín-Martín

**Affiliations:** 1 Faculty of Health Sciences, Department of Nursing, University of Málaga, Málaga, Spain; 2 Biomedical Research Institute of Málaga (IBIMA), Málaga, Spain; 3 Legal and Forensic Medicine Area, Department of Human Anatomy, Legal Medicine and History of Science, Faculty of Medicine, University of Málaga, Málaga, Spain

**Keywords:** Palliative care, Collett-Lester fear of death scale, Undergraduate, Validation, Occupational therapy, Legal medicine

## Abstract

**Background:**

The fear of death is a common experience among healthcare students and professionals that may impact the quality of care provided to patients, particularly those receiving palliative care. The Collett-Lester Fear of Death Scale is a widely used instrument to assess this fear, although its psychometric properties have not been extensively studied in Occupational Therapy students. The present study aimed to validate the Collett-Lester Fear of Death Scale (CL-FODS) in a sample of Occupational Therapy students and to explore its implications for palliative care education.

**Method:**

A cross-sectional study was conducted to perform psychometric testing of the CL-FODS in Occupational Therapy undergraduate students. Structural validity, internal consistency, and test-retest reliability were analysed. A total of 195 Occupational Therapy students were included in this study. Additionally, the participants completed a brief survey on their experiences and attitudes towards palliative care.

**Results:**

The internal consistency was satisfactory (α = 0.888). The exploratory factor analysis to evaluate the internal structure yielded four factors. The model fit indices were: comparative fit index = 0.89, and root mean square error of approximation = 0.06). The test–retest reliability was satisfactory and demonstrated an intraclass correlation coefficient of 0.939.

**Conclusion:**

The Spanish version of the CL-FODS showed satisfactory psychometric properties; therefore, assessing fear of death in Occupational Therapy students is helpful. This study highlights the importance of addressing fear of death and palliative care education in Occupational Therapy undergraduates to improve future professional attitudes and, consequently, the quality of patient care at the end of life.

## Background

In recent decades, mortality statistics show an increase in chronic degenerative diseases that lead to terminality [1, 2]. In Spain, there has been a rapid growth of palliative care (PC) programs and services in recent years in response to the high demand and the growing interest of the healthcare system in addressing it [3].

PC training is one of the lines of action of the World Health Organisation [4]. It has been considered an area for improvement in the PC Strategy of the Spanish National Health System [3] and the promotion of PC training programs continues to be a challenge. In this context, PC training must begin in the faculties and continue with postgraduate training and continuing education [5].

PC has great relevance during the training of health professionals, such as occupational therapists [6–8]. Caring for patients in a terminal situation requires theoretical knowledge and the development of a series of humanistic and emotional skills and competencies [9]. In this sense, the professional who works in the field of PC stands out for being a person with adequate training in psychology and an adequate attitude towards death [10].

The fear of death is a common experience among healthcare students and professionals. It has been associated with a range of negative outcomes, including burnout, compassion fatigue, and reduced quality of care provided to patients receiving PC [11]. As such, it is important to assess and address the fear of death among healthcare students, particularly those in fields such as Occupational Therapy, who are likely to encounter patients with life-limiting illnesses [12, 13]. In this regard, some studies have demonstrated a significant relationship between clinicians’ attitudes towards death and their approach to clinical work with dying patients [14].

Previous studies about fear of death showed gender differences, with women scoring higher on fear of death than men [15], and the most widely accepted explanation was that women have an easier time admitting and expressing their emotions and feelings related to death [16]. However, to date, these gender differences have not been clearly explained.

The training of health professionals at the undergraduate and postgraduate levels enables them to provide quality care and greater professional satisfaction [17]. It is also essential to identify the challenges a health professional in community PC must face and the competencies he/she should possess [18]. In this sense, there is a need to validate reliable and trustworthy tools to assess the impact of PC education in order to demonstrate the effectiveness of training programs [19].

End-of-life care teams must be interdisciplinary as the attention to the patient with these special needs is provided from a physical, psychological, social and spiritual approach [20]. In this sense, an ideal end-of-life care team should be composed of a physician, a nurse, an occupational therapist, a physiotherapist, a psychologist, and a social worker [21]. These professionals have different competencies and skills and their undergraduate training is different, so it is essential to validate the instruments separately [22]. In many cases, occupational therapy students need to undertake a specific care approach at the end of their degree training [8]. Professionally, they have direct contact with these patients and the development of their skills in daily practice is necessary to improve patient care by being patient-centred, promoting occupational engagement to optimize quality of life, involving the social and relational environment, enabling occupations and facilitating occupational adaptation [23, 24].

There is limited data on attitudes towards death among Occupational Therapy students since the literature on the measurement of this construct is relatively new. In this sense, we need tools that allow measuring this construct, providing greater evidence, since the evaluation of attitudes towards death is increasingly used in teaching, both in the clinical setting and in research [25–27].

The Collett-Lester Fear of Death Scale (CL-FODS) is one of the multidimensional classical instruments used in assessing attitudes towards death [28]. The original instrument contained 36-items, however it was revised to reduce it to 28-items in 2003. Conceptually it was designed to measure fear of death in four subscales: “Your Own Death”, “Your Own Dying”, “The Death of Others” and “The Dying of Others” [29] The questionnaire has also been validated in different languages such as Czech [30], French [15] or Arabic [31]. Although the CL-FODS has been adapted to Spanish in nursing students, to the best of our knowledge, no psychometric analysis has been carried out in Occupational Therapy students [32]. Thus, this study aimed to analyze structural validity, internal consistency, and test-retest reliability of the CL-FODS in Occupational Therapy undergraduate students.

## Materials and methods

### Design

A cross-sectional study was carried out to perform psychometric testing of the CL-FODS in Occupational Therapy undergraduate students. The established inclusion criteria were: (1) being a student of the Occupational Therapy degree, (2) ability to use mobile devices or computers, (3) cognitive ability to read and comprehend written text, and (4) signed informed consent. No exclusion criteria were applied.

The study was carried out at the Faculty of Health Sciences of the University of Malaga. Participants were recruited between January and June, 2022. The estimated sample size based on the enrollment of undergraduate students was 195 Occupational Therapy undergraduate students (1st, 2nd, 3rd and 4th year).

### Procedure

The questionnaires were distributed in electronic format through a QR code. The study and the questionnaire instructions were presented, while the QR code was offered to be scanned and completed in approximately 10/15 minutes. The objective and justification of the study, voluntary participation and the confidentiality of its results were explained. All participants accepted and signed the informed consent.

### Measures

#### The Collett-Lester Fear of Death Scale (CL-FODS)

The Spanish version of CL-FODS was used in this study [32]. This instrument consists of 28 items with five Likert-type response options from 1 (not) to 5 (very). The CL-FODS is divided into four differentiated subscales, which individually consist of 7 items, generating 28 items in total, which are: fear “Your Own Death “, “Your Own Dying”, fear of the “The Death of Others” and “The Dying of Others” [32]. The objective of this scale is to determine the level of stress or fear of the students in relation to these aspects. The total score varies from the minimum, which corresponds to 28 points (no fear regarding death), to 140 points, which corresponds to the maximum score (maximum degree of concern regarding death). Scores are obtained for the total scale and for each subscale, calculating the average of the respective answers. The highest mean scores indicate greater fear of death or of the dying process [32]. To improve the interpretation of the results, the total score was presented on a scale of 1 to 5, following Mondragon-Sanchez et al. [33].

### Data analysis

A descriptive analysis was performed to estimate the sociodemographic variables (sex, age, year of degree, previous experience with palliative care) and CL-FODS results. The description of the questionnaire results was based on the median and interquartile range due to their non-normal distribution according to the Shapiro-Wilk test. However, the results by sex, year and section of the questionnaire were provided based on the means and standard deviations in order to compare them with the results of other studies. The distribution results of CL-FODS were measured by the Shapiro-Wilk test. A one-way ANOVA with a post-hoc Scheffe test was performed to compare the mean and variance values of each subscale of CL-FODS, with sex as a fixed factor and grouped by year. For a non-parametric distribution, an ANOVA Kruskal-Wallis Test was applied. Statistical analysis was carried out to estimate the internal consistency, test-retest reliability and factor structure of the CL-FODS. Cronbach’s coefficients were calculated to obtain the internal consistency [34]. Intraclass correlation coefficient type 2.1 (ICC2.1) was calculated to examine the test-retest reliability [35]. The factor structure was determined using exploratory factor analysis (EFA) and confirmatory factor analysis (CFA).

Maximum Likelihood Extraction and varimax rotation were used in EFA. Items with a loading smaller than 0.4 or loadings on more than one factor were deleted. Kaiser–Meyer–Olkin (KMO) values and Bartlett’s test of Sphericity were used to assess the factorability of the data. A minimum ratio of five participants per item was required, as detailed in the literature [36]. The model fit indices included chi-square (χ²), the comparative fit index (CFI), and root mean square error of approximation (RMSEA) [37]. For CFI, values of 0.90 or above indicated a good fit. Values of 0.08 or below for RMSEA indicated a close fit [38]. The data analysis was carried out using SPSS version 24 and JASP version 0.16.3 [39, 40].

### Ethical aspects

The experimentation Ethics Committee of the University of Malaga approved the study (CEUMA registration number: 138-2021-H; November 4, 2021). This study complies with the principles established in the Declaration of Helsinki. Likewise, all the participants gave their consent, and all the data collected was treated anonymously for the present study.

## Results

A total of 195 Occupational Therapy undergraduate students were included in this study, and thus the ratio of patients per item was obtained. The mean age was 22.05 ± 6.19, and the distribution by sex was 30 men (15.4%) and 165 women (84.6%). Eighty of the 195 participants (41.0%) had previous palliative care experience. The questionnaire’s sum scores by cohort are shown in Table 1. The median value for CL-FODS was 3.79 (interquartile range: 3.36;4.18). The sample had a non-parametric distribution according to the Shapiro Wilk-test (*p* = 0.005). Male and female students ´ median value was 3.5 (2.96;3.77) and 3.8 (3.39;4.21), respectively.

**Table 1:** Descriptive sample value of CL-FODS by undergraduate year

	First	Second	Third	Fourth
	50 (25.6%)	52 (25.7%)	77 (39.5%)	16 (8.2%)
Median	3.79	3.89	3.86	3.29
Mean	3.71	3.80	3.78	3.44
SD	0.60	0.64	0.67	0.66
Minimum	2.00	2.29	1.96	2.25
Maximum	4.96	4.96	4.96	4.57
25th percentile	3.50	3.35	3.35	3.05
50th percentile	3.79	3.89	3.89	3.29
75th percentile	4.04	4.29	4.30	4.01

SD: standard deviation

Based on a previously described non-parametric distribution, an ANOVA test was performed for multiple Kruskal-Wallis comparisons between the average results of the CL-FODS by years (*p* = 0.25). The Independent Sample T-test (Mann-Whitney U test) showed significant differences by sex (*p* = 0.002), with a Hodges-Lehman Estimate of -0.429 at 95% Confidence Interval (-0.68; -0.18). In this sense, the individual analysis of each subscale by sex and undergraduate year based on one-way ANOVA post-hoc Scheffe showed significant differences. The subscale " Your Own Dying”, “The Death of Others” and " The Dying of Other”; women showed more fear of death and facing these situations than men in first year (Table 2). Comparison of fourth year should be taken with caution due to the small sample size of males, only 2 subjects.

**Table 2:** Differences in CL-FODS subscales by sex and by undergraduate year

Undergraduate year	CL-FODS Subscale	Male	Female	Mean Difference	SE	t	p_scheffe_
First	Your Own Death	2.79 ± 1.07	3.39 ± 0.80	-0.60	0.33	-1.84	0.07
	Your Own Dying	3.20 ± 0.98	3.86 ± 0.67	-0.66	0.28	-2.38	0.02
	The Death of Others	3.34 ± 0.83	4.26 ± 0.48	-0.92	0.21	-4.38	˂0.001
	The Dying of Others	2.75 ± 0.64	3.83 ± 0.51	-1.08	0.20	-5.29	˂0.001
Second	Your Own Death	3.33 ± 0.51	3.33 ± 1.08	-0.01	0.42	-0.02	0.99
	Your Own Dying	3.53 ± 0.36	3.99 ± 0.65	-0.46	0.25	-1.82	0.07
	The Death of Others	3.84 ± 0.72	4.25 ± 0.71	-0.41	0.29	-1.44	0.16
	The Dying of Others	3.57 ± 0.60	3.76 ± 0.78	-0.19	0.31	-0.61	0.58
Third	Your Own Death	3.09 ± 1.10	3.36 ± 1.07	-0.27	0.33	-0.83	0.41
	Your Own Dying	3.68 ± 0.63	3.92 ± 0.72	-0.24	0.21	-1.10	0.27
	The Death of Others	4.00 ± 0.60	4.25 ± 0.61	-0.25	0.19	-1.33	0.19
	The Dying of Others	3.45 ± 0.91	3.79 ± 0.77	-0.34	0.24	-1.42	0.16
Fourth	Your Own Death	1.64 ± 0.51	3.21 ± 0.71	-1.57	0.53	-2.97	0.01
	Your Own Dying	2.93 ± 0.91	3.73 ± 0.90	-0.81	0.68	-1.18	0.26
	The Death of Others	4.29 ± 0.61	3.66 ± 0.76	0.62	0.57	1.09	0.29
	The Dying of Others	2.86 ± 0.20	3.46 ± 0.74	-0.60	0.54	-1.12	0.28

*p* = 0.05

### Structural validity and internal consistency

The Kaiser-Meyer-Olkin value was 0.866, and Bartlett’s Test of Sphericity was significant (*p* < 0.001). This indicates that the common variance was adequate, providing support for the use of EFA for this data set. Up to 42.6% of the variance could be explained with four factors, with an eigenvalue higher than 1.

In this factor analysis, items 8, 9,14, 20, 22 and 23 did not significantly load in the proposed factor and load in different factors (Table 3). These items were considered as “problematic items” and deleted from the analysis. In the case of factor 1, the item with the highest factor loading was item 6 (0.688); for factor 2, item 21 (0.743); for factor 3, item 25 (0.977); and for factor 4, item 10 (0.765) (Table 3).

**Table 3:** Factor loading and Internal Consistency of the Collett-Lester Fear of Death Scale

	Factor Loading				Internal Consistency	
Items	Factor 1	Factor 2	Factor 3	Factor 4	Corrected item-total correlation	Cronbach’s alpha if item deleted
Item 1	**0.467**	0.083	0.028	0.085	0.476	0.917
Item 2	**0.620**	0.263	0.018	0.146	0.647	0.914
Item 3	**0.604**	0.085	0.082	0.106	0.566	0.916
Item 4	**0.552**	0.179	0.001	0.127	0.541	0.916
Item 5	**0.558**	0.135	-0.025	0.034	0.511	0.917
Item 6	**0.688**	0.139	0.056	0.076	0.593	0.915
Item 7	**0.551**	0.062	0.042	0.028	0.504	0.917
Item 8	0.404	-0.009	0.102	0.358	0.456	0.917
Item 9	0.296	0.227	0.141	0.369	0.487	0.917
Item 10	0.167	0.276	0.040	**0.765**	0.486	0.917
Item 11	0.159	0.191	0.197	**0.690**	0.483	0.917
Item 12	**0.652**	0.168	0.142	0.065	0.599	0.915
Item 13	**0.634**	0.155	0.162	0.241	0.626	0.914
Item 14	0.261	0.324	0.128	0.156	0.470	0.917
Item 15	0.068	**0.579**	0.118	0.268	0.496	0.917
Item 16	0.203	**0.461**	0.114	0.061	0.425	0.918
Item 17	0.120	**0.561**	0.215	0.247	0.514	0.917
Item 18	0.127	**0.612**	0.110	-0.034	0.443	0.917
Item 19	0.227	**0.659**	0.140	0.137	0.582	0.916
Item 20	0.255	0.215	0.198	0.099	0.404	0.918
Item 21	0.183	**0.743**	0.158	0.210	0.599	0.915
Item 22	0.200	0.255	0.352	0.045	0.499	0.917
Item 23	**0.432**	0.222	0.241	-0.118	0.524	0.916
Item 24	0.046	0.340	**0.549**	0.186	0.440	0.917
Item 25	0.109	0.156	**0.977**	0.061	0.500	0.917
Item 26	0.282	**0.545**	0.175	0.051	0.599	0.915
Item 27	-0.027	0.343	**0.537**	0.316	0.487	0.917
Item 28	**0.565**	0.325	0.087	0.194	0.685	0.914

Rotation Method: Varimax.

**Fig. 1:**
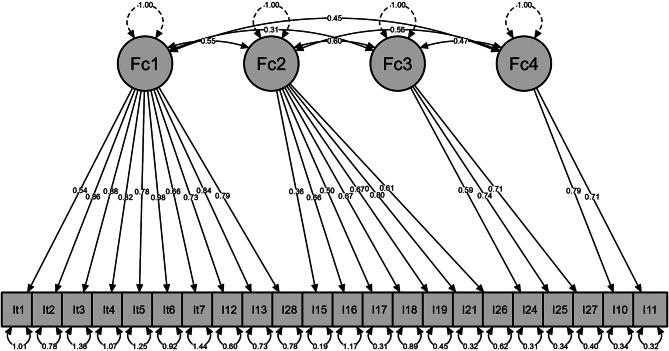
Results of Fit index, RMSA and CFI values of Collett-Lester Fear of Death Scale. Note:χ²=372.502 (df = 203, *p* < 0.001), CFI = 0.89 and RMESA = 0.06

The fit indices of the four-factor model indicated a good fit for RMESA (RMESA = 0.06), and the CFI value was close to 0.9. Chi square value was χ²=372.502 (df = 203, *p* < 0.001) (Fig. 1).

Of the 28 items in the CL-FODS questionnaire, the two items with the highest corrected item-total correlation were: item 2 (0.647) and item 28 (0.685). The overall internal consistency of the final questionnaire (22 items) was 0.888. The final 22 items CL-FODS presented good internal consistency to factor 1 (α = 0.861), factor 2 (α = 0.818), factor 3 (α = 0.792) and factor 4 (α = 0.771).

### Test-retest reliability

To evaluate this measurement property, 69 participants completed the CL-FODS twice, separated by 15 days. Test-retest reliability of the 22 items CL-FODS was demonstrated, with an ICC of 0.939.

## Discussion

The present study provides a descriptive analysis of the results obtained in the CL-FODS questionnaire as well as a psychometric analysis of its validity and reliability for students of Occupational Therapy. To our knowledge, this study is the first psychometric analysis of the CL-FODS in Occupational Therapy students. The psychometric testing of the CL-FODS was carried out satisfactorily, showing adequate values for internal consistency, structural validity, and test-retest [34–36]. The attitudes towards death are difficult to evaluate and define [25]; however, validated measures for assessing this construct can be useful in teaching, research and the clinical setting.

In order to compare with a similar research in a population of nursing students (112.2 ± 18.6) [30], the total score of our study was converted to a range of 28 to 140 points; the result was 104.03 ± 16.576, indicating a lower level of fear of death. This small difference could be explained by the demographic characteristics of the sample (only women nursing including bachelor and master degree) and the differences in the university education of both degrees.

A previous study carried out on a French population showed that women have a greater fear of death than men, more specifically in the subscales “Your Own Dying” and “The Death of Others” [15]. Gender differences were also observed in nursing students [16] in the subscales of the CL-FODS, in the subscales of “Your Own Death " in the first-, second- and third-year students, and in the subscale of “The Death of Others” in the first- and second-year students, with women having more fear of death than men. Likewise, the resilience of these students was evaluated through the Resilience Scale (ER-14), with males in the third year showing greater resilience [16]. As can be observed in Table 2, women showed more fear of death according to the CL-FODS questionnaire in the subscales “Your Own Death”, “The Death of Others” and “The Dying of Others” regardless of the degree year. These differences were minimized when the degree year was included in the analysis (Table 2).

Regarding structural validity, the data were adequate for factor analysis. The EFA carried out to evaluate the internal structure yielded four factors. CFA was performed using the four-factor model and showed good fit for RMESA (0.06) [37]. The CFI value was close to 0.9 [38]. In this model, we removed items 8, 9, 14, 20, 22 and 23, and we developed a shorter version of 22 items. Thus, the findings from the EFA and CFA indicated a four-factor structure of the CL-FODS, providing support for construct validity. The four-factor structure of the CL-FODS has been reported in previous studies [15, 30]. However, other studies have revealed a five-factor structure for this instrument [41]. In this regard, the validation and cross-cultural adaptation to Spanish in the nursing population were aslo adequate for an EFA analysis, as they obtained a satisfactory result in the Kaiser-Mayer-Olkin test (KMO = 0.982) [32]. However, the EFA analysis with a Rotated Varimax Factor Structure provided a four-factor solution that explained 51.9% of the total variance, which did not maintain the original distribution of items into factors [32].

The Czech version identified four components that could explain 50.5% of the variance. This factor solution did not maintain the original distribution of items into factors [30]. The EFA analysis performed on the French version of the questionnaire is also justified. On a sample of 207 participants, the KMO measure was 0.88 and Bartlett’s test of Sphericity was significant, with χ2 (378) = 2811; *p* < 0.001 [15]. These results are in agreement with those obtained in the present study, in which KMO = 0.866 on a sample of 195 participants. In line with the results obtained by the Czech version, the French version also obtained a 4-factor distribution, explaining 43.5% of the variance. The distribution of the factors is similar to that of the original subscales of the questionnaire. However, it identifies items 12, 14, 16, 20, and 28 with loading values below 0.50 [15]. Based on these results, the study published by Cuniah et al. [15] suggests a revised model with a reduction in the number of items, due to the possibility of overlapping information, maintaining the good psychometric properties of the questionnaire.

The internal consistency was satisfactory (α = 0.888), which indicates a good homogeneity of the items [28]. These values are in line with those reported in other studies (Cronbach’s α = 0.914) [30, 32]. In this sense, the Czech adaptation of the CL-FODS reported a lower value (Cronbach´s α = 0.75), and intercorrelations of the different subscales ranged from 0.451 to 0.586 [30]. Moreover, the previous Spanish version showed good internal consistency (Alpha coefficient: 0.79–0.86); these results were higher than the test-retest results obtained in the same study [32].

The test-retest reliability was satisfactory, and it was demonstrated with an ICC of 0.939, providing reliability evidence [35]. This value was higher than those reported in other studies [32, 41]. The Czech adaptation of the CL-FODS reported an internal reliability of 0.914; this reflects 9% random oscillations [30]. In this way, Tomás-Sábado et al. obtained acceptable-to-good test-retest results after two weeks for each of the four sections of the questionnaire, with values between 0.72 and 0.82 in a sample of 281 participants (109 nursing professionals and 172 nursing students) [32].

Previous studies have also performed correlation analyses with questionnaires with similar characteristics to assess convergent and discriminant validity. Tomás-Sábado et al. [32] compared the results of the CL-FODS questionnaire with Death’s Anxiety Inventory (DAI) and the Kuwait University Scale (KUAS). All observed correlations were positive and significant (*p* < 0.01), showing a stronger correlation with the DAI. Likewise, Pearson’s correlation scores ranged from 0.50 to 0.62 according to the CL-FODS subscale with the DAI (strong correlation), and 0.36 to 0.42 with the KUAS (moderate correlation). In this context, the lower correlations between the CL-FODS subscales and general anxiety (KUAS), compared to death anxiety (DAI), provided support for discriminant validity. Convergent validity of the CL-FODS with the DAS was evaluated in the French version, obtaining strong correlations with the “Your Own Death” subscale (*r* = 0.65) and moderate with the “Your Own Dying” (*r* = 0.41), “The Death of Others” (*r* = 0.39) and “The Dying of Others” (*r* = 0.39) subscales [15].

### Clinical implications

This study is relevant to address the fear of death of Occupational Therapy students and palliative care training to enhance the attitudes of future professionals. This may improve the quality of care for patients at the end of life. Clemens et al. 2021 studied the impact of a 12-hour training course in empathy, attitude and fear of death with Kiersma-Chen Empathy Scale, Frommelt Attitudes Toward Care of the Dying Scale Form B and CL-FODS, respectively [42]. Significant differences were observed for all three instruments used (*p* < 0.01) [42]. A longitudinal study on 100 nursing students during the three years of training showed significant differences in the subscale “Your Own Dying” of the CL-FODS instrument. They observed that nursing students in the three academic years feared the “Death of Others” the most, followed by “Dying Itself” and their “Own Death” [16]. Students showed more fear in the scale “Your Own Dying” in the third year than in the first year [16], which could be explained by the fact that the training is mainly focused on the process of “Death of others”. Both studies demonstrated the importance of the training process and the acquisition of skills [16, 42]. Therefore, it is necessary to increase the specific training that students receive in relation not only to PC, but also to coping with death since it is scarce.

This study has international applicability; the CL-FODS in Spanish Occupational Therapy students could also assess the fear of death in Spanish-speaking countries (Spain and Latin America) and in countries with a large Spanish-speaking population, such as the USA. In these cases, it would be important to carry out a cross-cultural validation prior to its use, modifying some linguistic aspects and identifying differences in the undergraduate programs.

### Study limitation

This is the first study to carry out psychometric testing of the CL-FODS in Spanish Occupational Therapy students. In this regard, the psychometric analysis of this measure was performed satisfactorily. One limitation of our study is the sample size. A larger sample size would allow performing a confirmatory factor analysis with greater precision. Further research should include a second reference questionnaire as a “gold standard” in order to assess the construct validity.

## Conclusions

This study is the first psychometric analysis of the CL-FODS in Spanish Occupational Therapy students. Evidence for the reliability and validity of the CL-FODS is provided in the present study. Thus, this is a valid and reliable measure for assessing attitudes towards death in Occupational Therapy students. Although no significant differences were observed between the different years compared to the CL-FODS questionnaire, previous studies have shown that training effectively improves these attitudes. The unit of analysis did not undertake specific training in end-of-life care during their undergraduate training. Additional research in a larger sample is needed to develop a full psychometric validation and strengthen the data obtained.

## Data Availability

The datasets used and/or analysed during the current study are available from the corresponding author on reasonable request.

## Abbreviations

CL-FODS: Collett-Lester Fear of Death Scale
PC: palliative care
ICC: Intraclass correlation coefficient
EFA: exploratory factor analysis
CFA: confirmatory factor analysis
KMO: Kaiser-Meyer-Olkin
CFI: comparative fit index
RMSEA: root mean square error of approximation
ER-14: Resilience Scale
DAI: Death’s Anxiety Inventory
KUAS: Kuwait University Scale
